# Are berries useless by-products of ginseng? Recent research on the potential health benefits of ginseng berry

**DOI:** 10.17179/excli2017-376

**Published:** 2017-05-22

**Authors:** Tae Kyung Hyun, Keum-Il Jang

**Affiliations:** 1 Department of Industrial Plant Science and Technology, College of Agricultural, Life and Environmental Sciences, Chungbuk National University, Cheongju 28644, Republic of Korea; 2 Department of Food Science and Biotechnology, College of Agricultural, Life and Environmental Sciences, Chungbuk National University, Cheongju 28644, Republic of Korea

## ⁯

Dear Editor,

Since the ginseng root (*Panax ginseng* C. A. Meyer) has long been used as a valuable medicinal plant in traditional oriental medicine, current pharmaceutical studies have sought to reveal its other potential applications. These include a wide array of ameliorative effects that encompass those for anti-oxidation, anti-inflammatory, antihistamines, anti-obesity, anti-diabetic, anti-tumor, enhancing immune system function, adjusting blood pressure, sexual potentiation and so on (Li and Gong, 2015[19]; Kim et al., 2016[12]; Patel and Rauf, 2017[22]; Zhang et al., 2017[27]). 

When culturing ginseng, cultivators are required to choose between harvesting the seed for further plantings or removing the inflorescences to increase root development (Fiebig et al., 2005[5]), which suggests that the ginseng berry (fruit) may be considered a useless by-product of ginseng. However, phytochemical analyses determined that ginseng berries contained higher amounts of total ginsenosides than the root (Kim et al., 2009[16]). In addition, ginsenoside Re, a major constituent of the ginseng berry, exhibited multiple pharmacological activities including anti-diabetic, anti-inflammatory, anti-oxidation, neuroprotective, anti-arrhythmic and anti-ischemic effects, as well as supporting osteoblast differentiation and cardiovascular health (Chen et al., 2008[1]; Lee et al., 2012[18]; Peng et al., 2012[23]; Kim et al., 2017[10]; Huang et al., 2016[7]; Kim et al., 2017[13]). These findings indicate the potential of ginseng berries as beneficial biomaterials for the food and medical industries; however, ginseng berries have long been underappreciated. 

To introduce the ginseng berry as a potential source of herbal medicine, we summarized key findings that demonstrate the pharmacological properties of ginseng berries (Table 1(Tab. 1); References in Table 1: Yang et al., 2014[26]; Kim et al., 2012[15]; Park et al., 2012[21]; Seo et al., 2015[24]; Choi et al., 2017[3]; Cho et al., 2013[2]; Choi et al., 2013[4]; Zhang et al., 2016[29]; Kim et al., 2012[9]; He et al., 2016[6]; Wang et al., 2015[25]; Zhang et al., 2015[27]; Jung et al., 2016[8]; Kim et al., 2016[11]; Kim et al., 2012[9]; Kim et al., 2017[14]; Park et al., 2015[20]; Lee et al., 2014[17]). This report also emphasizes the potential of ginseng berries to be employed in new herbal medicine, and we hope that this report will stimulate future research on the ginseng berry for its applications in the pharmaceutical industry.

## Notes

Tae Kyung Hyun and Keum-Il Jang (Department of Food Science and Biotechnology, College of Agricultural, Life and Environmental Sciences, Chungbuk National University, Cheongju 28644, Republic of Korea; Phone: +82-43-261-2569, Fax: +82-43-271-4412, E-mail: jangki@chungbuk.ac.kr) contributed equally as corresponding authors.

## Conflict of interest

The authors declare no conflict of interest.

## Figures and Tables

**Table 1:**
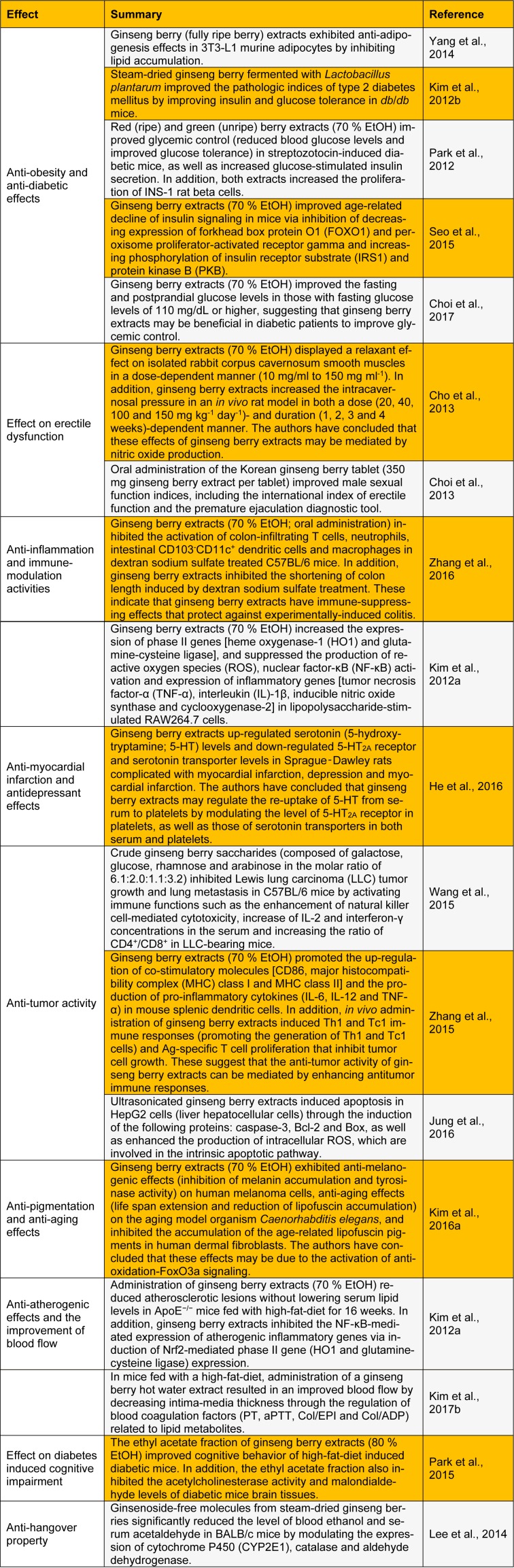
Recent studies on the biological and pharmacological activities of ginseng berries
